# War‐Related Disruptions and Health Service Outcomes Among Internally Displaced Persons in Tigray, Ethiopia: Evidence From Adigrat City

**DOI:** 10.1002/puh2.70288

**Published:** 2026-06-01

**Authors:** Fikre Belay Tekulu, Haftom Teshale Gebre, Meseret Meresa Weldemichael, Esayas Weldetinsae Gebrekidan

**Affiliations:** ^1^ Department of Geography and Environmental Studies, College of Social Science and Humanities Adigrat University Adigrat Ethiopia; ^2^ Department of Management, College of Business and Economics Adigrat University Adigrat Ethiopia; ^3^ Department of Mathematics College of Natural Science and Computation Adigrat University Adigrat Ethiopia

**Keywords:** Adigrat, Ethiopia, health services, internally displaced persons (IDPs), Tigray, war

## Abstract

Internally displaced persons (IDPs) are individuals or groups forced to leave their homes due to conflict, violence, or other coercive circumstances. This study examined the effects of armed conflict on health service availability and outcomes for IDPs residing in designated centers in Adigrat City, Tigray region, Ethiopia. From a total of 13,315 households (HHs), a stratified random sample of 373 respondents was drawn across four zones of Tigray. Key informants were selected using purposive and simple random sampling. Data were collected through direct observation, structured questionnaires, and interviews with key informants. The study focused on key indicators of health service disruption, including destruction of health facilities, access to health extension services, and treatment for chronic diseases, as well as broader health outcomes such as maternal, infant, and elderly mortality. Findings revealed severe negative consequences of war, including widespread destruction and collapse of hospitals and health centers. Regression analysis indicated that approximately 69.8% of the disruption in health service delivery and infrastructure within IDP areas was associated with the combined effects of disrupted banking services and increased crime rates resulting from the conflict. Overall, the study underscores the urgent need for coordinated postwar reconstruction, restoration of critical systems, and targeted interventions to rebuild the health sector and improve the well‐being of displaced populations in Tigray.

## 1 Introduction

Conflict and violent crises have resulted in over 40 million internally displaced persons (IDPs) globally as of 2020 [1]. Millions of people worldwide are impacted by armed conflict, which seriously impedes efforts to achieve the United Nations Sustainable Development Goals (UN SDGs) [2, 3]. Armed conflicts negatively affect health both directly through violence and indirectly through socioeconomic instability. Major health effects include trauma, mental health conditions, noncommunicable diseases, child health problems, sexual and reproductive health challenges, maternal health hazards, and a rise in infectious diseases [4, 5]. According to Garry and Checchi [4], vulnerable groups such as women, children, newborns, and older adults are disproportionately affected and require additional care. Preexisting demographic issues and accessibility of healthcare services further influence health outcomes in conflict settings.

In Africa, prolonged conflicts and internal displacement have created severe public health challenges. Somalia, for example, hosts a large IDP population affected by recurrent natural disasters and conflict, facing limited healthcare access, poor living conditions, and high rates of preventable diseases [5]. In 2023, an estimated 3.86 million Somalis were internally displaced, making Somalia one of the countries with the largest IDP populations globally [5]. Across Africa, women in particular face new health challenges due to armed conflict and displacement [6]. Similarly, the ongoing armed conflict in Sudan, which began on April 15, 2023, has severely affected the nation's healthcare system, exacerbating existing vulnerabilities and creating new public health challenges [7]. In Sudan, ongoing conflict has left IDPs struggling to access healthcare services. Among 612 surveyed IDPs, only 33.6% reported consistent access to qualified staff, 22.8% visited public healthcare facilities supported by NGOs, and more than half found healthcare unaffordable [8]. Healthcare personnel have faced significant risks, limiting their ability to deliver essential services, whereas medical facilities have experienced operational declines. Vulnerable populations, including women, children, and displaced persons, have been disproportionately affected [9].

According to Ayele [10], armed conflict in Ethiopia has severely affected nutrition, healthcare access, and overall health outcomes. The war and siege in Tigray (November 2020–November 2022) caused massive internal displacement and led to the destruction or dysfunction of more than three‐quarters of the region's health facilities due to damage and looting [WHO] [11]. In Mekelle City, a major urban center hosting large numbers of displaced people, 11 IDP clinics were established to provide services to 6732 patients, including 3465 men [12]. According to WHO [11], by March 2021, only 22% of health centers in Tigray were operational due to power cuts and ongoing hostilities, leaving nearly 80% inaccessible. Referral, general, and primary hospitals in the region also suffered significant impairments [13].

War is commonly defined as openly declared armed conflict between nations or states; however, many conflicts begin without official declarations. Legal recognition of war is determined by formal declarations, although hostilities may occur prior, and is generally characterized by principles of jus ad bellum [14]. Wars have detrimental effects on human life and economic infrastructure [15]. Human rights treaties, including Article 12 of the International Covenant on Economic, Social, and Cultural Rights (ICESCR), guarantee the right to health and emphasize equitable access to health facilities, goods, and services, particularly for vulnerable populations [16].

Although prior studies have documented the health impacts of conflict and displacement in Africa and Ethiopia, few have specifically examined how the Tigray War affected healthcare access for IDPs in Adigrat City. This study addressed this gap by assessing the impacts of war and humanitarian interventions on health services for IDPs residing in IDP centers in Adigrat, Tigray, Ethiopia. The objective is to understand how the war influenced healthcare access, delivery, and outcomes for displaced populations in this setting.

### 1.1 Research Questions

This study was guided by the following research questions:

1. How has the war in Tigray affected the provision and quality of health services for IDPs in Adigrat City?
2. What sociodemographic and contextual factors influence the level of disruption to health services in IDP centers?
3. What are the key health outcomes among IDPs in Adigrat City, and how are these outcomes associated with disruptions in healthcare services?

### 1.2 Theoretical Framework

**FIGURE 1:**
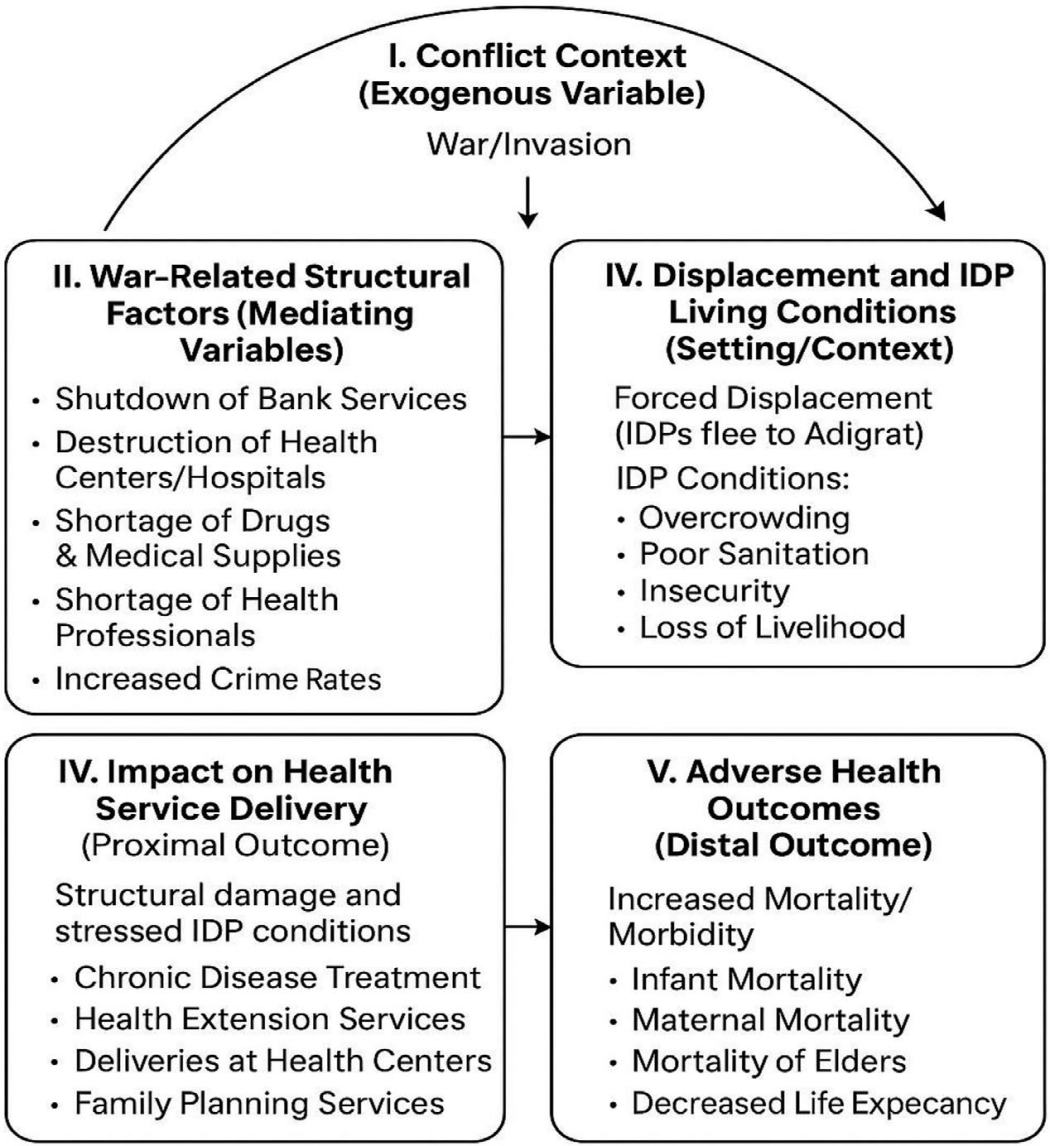
Theoretical framework. IDP, internally displaced person.

This study draws on key concepts from war theory and ecological systems theory to analyze the impact of armed conflict on health services for IDPs in Adigrat City, Tigray region, Ethiopia. Integrating these frameworks provides a multidimensional perspective on how conflict affects health service provision in IDP centers. “Theoretical Framework” on affects health service provision in IDP centers (see Figure 1).

### 1.3 War Theory

War theory, rooted in Karl Marx's analysis of class conflict and structural inequality, conceptualizes conflict, including war, as arising from material contradictions and social tensions [17, 18, 19]. Subsequent scholars have emphasized that wars evolve through cycles of negotiation, dissent, and accommodation, shaping societies and institutions over time [20, 21, 22, 23]. Modern conflicts, including the Tigray war, are often driven by identity politics, involve multiple armed actors, rely on informal war economies, and have severe civilian consequences, such as displacement and the disruption of essential services [24]. In this context, war theory helps explain how political, economic, and social factors converge to create instability, exacerbate disparities, and hinder access to health services for displaced populations.

### 1.4 Ecological Systems Theory

Ecological systems theory, proposed by Bronfenbrenner [25], posits that human development is shaped by interactions across multiple levels of social environments: microsystem, mesosystem, ecosystem, microsystem, and chronosystem. In IDP centers, the war disrupts these interconnected systems, leading to resource scarcity, fractured support networks, and weakened institutional aid. Applying ecological systems theory highlights that such disruptions are not isolated but reverberate across broader social systems. This perspective underscores the importance of considering contextual and systemic factors when evaluating the effects of war on health services. It also informs the design of holistic interventions to support IDPs effectively. The following Figure 1 illustrates the affects health service provision in IDP centers.

## 2 Materials and Methods

### 2.1 Study Area and Population

#### 2.1.1 Location

**FIGURE 2:**
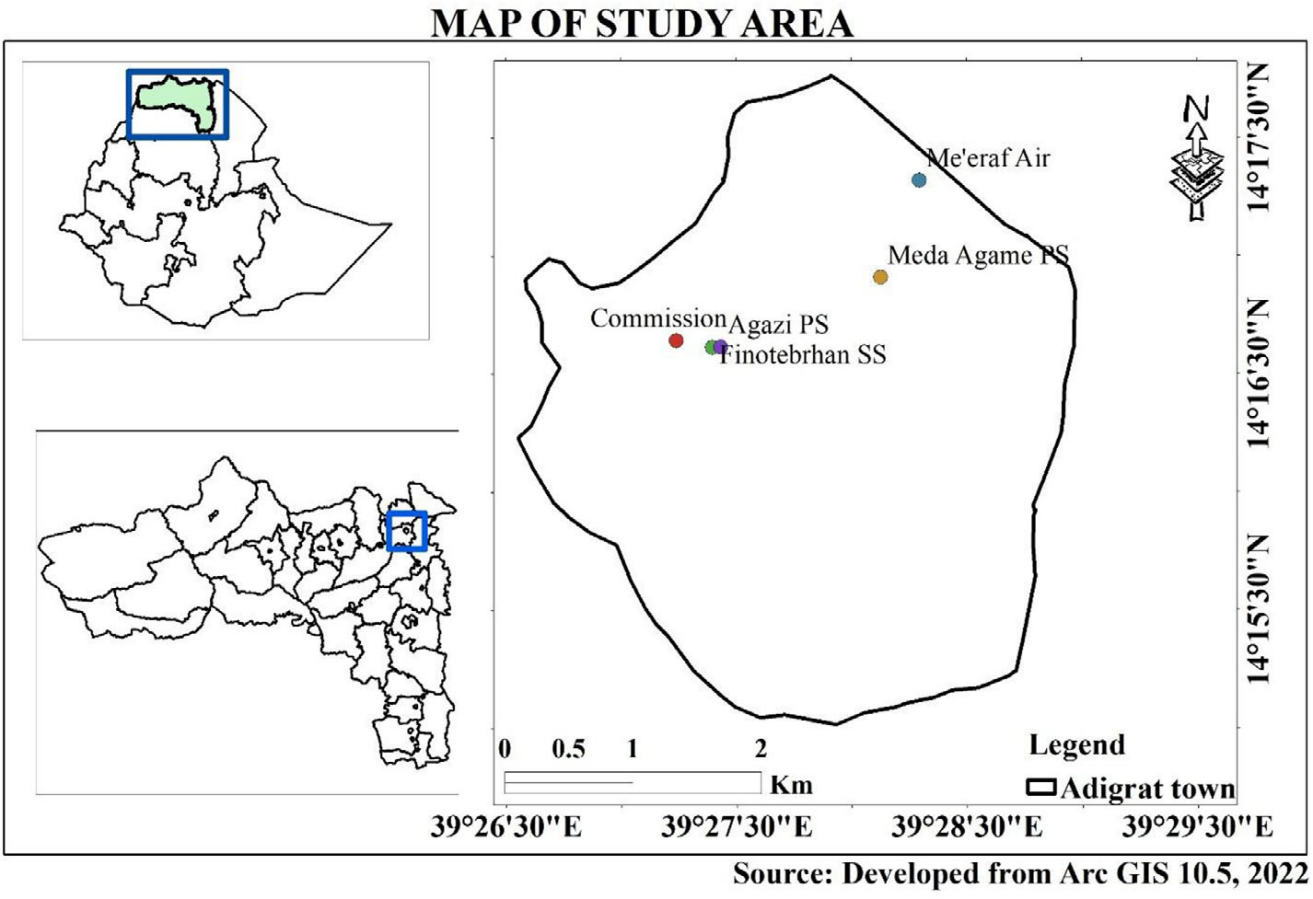
Map of study area.

Adigrat City, located in the eastern zone of Tigray, Ethiopia, hosts IDPs from the western, northwestern, central, and eastern zones of the region. The city currently hosts approximately 60,000 IDPs across the commission, Me'eraf Air, Agazi Primary School, Meda Agame Primary School, and Finotbrahan Secondary School. Tables 1 and 2 provide a breakdown of IDPs by household (HH) and individual, respectively (Figure 2) [26, 27].

**TABLE 1:** Number of internally displaced person (IDP) household heads by geographic zone.

No.	IDPs per household head	Male	Female	Total	Percent
1	Central zone	546	576	1122	8.43
2	Northwestern	616	448	1064	7.99
3	Western	2444	1365	3809	28.61
4	Eastern	3265	4055	7320	54.98
Total HHs		6871	6444	13,315	100.0

Abbreviation: HHs, households.

*Source:* Social affair office, 2022.

**TABLE 2:** Number of individual internally displaced persons (IDPs) by geographic zone.

No.	IDPs per individual	Male	Female	Total	Percent
1	Northwestern	3136	3126	6262	10.53
2	Central zone	2570	2658	5228	8.790
3	Western	9742	9626	19,368	32.56
4	Eastern	13,513	15,104	28,617	48.12
IDPs per individual		28,961	30,514	59,475	100.0

*Source:* Social affair office, 2022.

Table 1 presents the distribution of IDP HH heads by geographic zone.

Table 2 shows the total number of individual IDPs across the same zones.

IDPs in Adigrat have been forced to flee their homes following the outbreak of war. Currently, these IDPs are being hosted in several locations within the city, including Commission, Me'eraf Air, Agazi Primary School, Meda Agame Primary School, and Finotbrahan Secondary School. According to the Social Affairs Office of Adigrat City [28], approximately 60,000 IDPs are residing in these sites shown on Table 2.

### 2.2 Methodology

#### 2.2.1 Research Design

A descriptive cross‐sectional survey design was employed to capture the impact of the war on health services at a single point in time. This approach allowed for a representative description of the views and experiences of IDPs in Adigrat City.

#### 2.2.2 Target Population

The study targeted HH heads residing in IDP centers who were at least 18 years old, had lived in the center for a minimum of 3 months, and provided informed consent to participate.

#### 2.2.3 Sample Size and Sampling Technique

A sample of 373 HH heads was selected from a total of 13,315 registered HHs using stratified and simple random sampling. Strata were based on the four zones of origin. Stratification ensured proportional representation, and simple random sampling minimized selection bias. Non‐probability purposive sampling was used to select IDP centers with the highest number of affected individuals. The sample size was calculated using Kothari's [29] formula assuming a 95% confidence level and 5% margin of error:

$$n=\frac{Nz^{2}PQ}{E^{2}\left(N-1\right)+z^{2}PQ}$$

$$n=\frac{13,315{\left(1.96\right)}^{2}\left(0.5\right)\left(0.5\right)}{{\left(0.05\right)}^{2}\left(13,315-1\right)+{\left(1.96\right)}^{2}\left(0.9\right)\left(0.1\right)}$$

n=373

#### 2.2.4 Data Collection Instruments

##### 2.2.4.1 Questionnaire

A structured questionnaire was used to collect quantitative data on demographics, causes of displacement, and the social effects of displacement. A composite health services and infrastructure index was calculated from 12 Likert‐scale items (1 = very low disruption, 5 = very high disruption; Cronbach's *α* = 0.91).

##### 2.2.4.2 Key Informant Interviews (KIIs)

Narrative data were collected through KIIs with 12 purposively selected participants, including social affairs officers, caseworkers, and health experts in Adigrat. They were asked to what extent the health institutions are damaged and how the IDPs are suffering from a lack of medicines and other health provisions in IDP centers. All interviews were conducted in Tigrigna, the local working language, to ensure clarity and accurate expression of participants’ perspectives. Pseudonyms (e.g., KII‐health expert) were used to maintain confidentiality. The KIIs were conducted by trained data collectors with backgrounds in social science and public health. Prior to data collection, the interviewers received training on ethical considerations, interview techniques, and note‐taking procedures.

In addition, structured survey data were collected from 373 HH heads residing in IDP sites. The survey included questions on demographic characteristics, causes of displacement, and social impacts of displacement, in addition to the 12‐item health services and infrastructure index. The index items were completed by the HH respondents as part of the structured questionnaire. Data were collected at multiple IDP hosting sites within Adigrat City, including schools and temporary shelters where displaced populations were residing at the time of the study.

##### 2.2.4.3 Direct Observation

The researchers conducted field observations as a participant in the study area. This involved observing the living conditions and environments of IDP centers to gather relevant information about health service delivery and infrastructure impacted by the war. Direct observation was crucial in providing firsthand insights into the everyday lives of IDPs.

##### 2.2.4.4 Secondary Data

Secondary data were collected from published and unpublished documents, journal articles, reports, and online sources to provide background information and contextual understanding of the study area and war situation in Adigrat.

The health services and infrastructure index, which served as the dependent variable, were constructed as a composite measure of the level of disruption and damage to health services and infrastructure in IDP settings. The index was developed using responses from the HH survey of IDP heads, based on 12 Likert‐scale items assessing health service disruption (1 = very low disruption to 5 = very high disruption) (Table 3).

**TABLE 3:** Description of study variables and measurements.

Variable type	Variable	Measurement/Description
Dependent	Disruption of health services	Composite score from 12 Likert items assessing disruption levels
Independent	Sociodemographic factors	Age, sex, household size, and zone of origin
Moderating	Duration of displacement	Time spent in IDP center

Abbreviation: IDP, internally displaced person.

#### 2.2.5 Data Analysis

##### 2.2.5.1 Quantitative Analysis

The quantitative data were coded and analyzed using SPSS version 20. Descriptive statistics, including frequencies, percentages, means, and standard deviations, were computed to summarize the data. Inferential statistics were applied to examine the marginal effects of independent variables on the level of health service disruption.

##### 2.2.5.2 Qualitative (Narrative) Analysis

The KIIs were transcribed and analyzed using narrative analysis. Systematic coding was conducted to identify patterns and themes related to health service disruption, and two independent coders were employed to ensure inter‐coder reliability.

##### 2.2.5.3 Data Analysis Process

The data analysis process involved several steps. First, all data were checked, edited, and coded for accuracy. Quantitative data were then analyzed using SPSS, whereas narrative data from KIIs were examined through narrative analysis. Finally, triangulation across questionnaires, KIIs, and direct observations was conducted to enhance the validity and credibility of the findings.

## 3 Results

The study involved IDPs in Adigrat City with diverse sociodemographic backgrounds. Of the 373 respondents, 48.3% were female, 60.87% were married, and the majority (54.74%) were under the age of 31–40. The average age of respondents was 31–40 years. The length of time they had been in the IDP camps was 24 months.

### 3.1 Displacement Patterns and Dynamics

As indicated in Table 4, the findings indicate widespread displacement, particularly urban–urban migration (mean = 4.85), suggesting that IDPs moved between urban centers in search of safety. Rural–urban displacement was also high (mean = 4.36), reflecting large‐scale movement from conflict‐affected rural areas. Narrative evidence supports these findings, with respondents describing displacement as a survival strategy driven by insecurity, violence, and lack of basic services.

**TABLE 4:** Trends of displacement among internally displaced persons (IDPs) in Adigrat City.

Displacement type		Cases in percent and frequency					Mean	SD
		VL	L	M	H	VH		
Incidence of rural–urban displacement	*F*	14	12	38	72	237	4.36	1.039
	%	3.8	3.2	10.2	19.3	63.5		
Incidence of urban–urban displacement	*F*	0	0	9	38	326	4.85	0.420
	%	0.0	0.0	2.4	10.2	87.4		

Abbreviation: *F*, frequency.

As indicated on Table 4, this situation, indicating that alliance forces’ offenses and atrocities against civilians in Tigray resulted in more than 19 displaced people dying due to lack of food and medication, deliberate bombings, and the burning of health facilities, was further supported by a KII‐health expert who noted, “The destruction of the main hospital meant all complex cases were untreatable, leading to unnecessary deaths, even in routine maternity cases.”

This situation indicates that alliance forces’ offenses and atrocities against civilians in Tigray resulted in more than 19 displaced people dying due to lack of food and medication, deliberate bombings, and the burning of health facilities. This outcome was further supported by KIIs with a social affairs officer, who stated: “We saw people dying because of a lack of basic medication, not just violence. When the banks shut down, aid could not reach us, and the health centers that were not burned were left without staff or supplies.”

**FIGURE 3:**
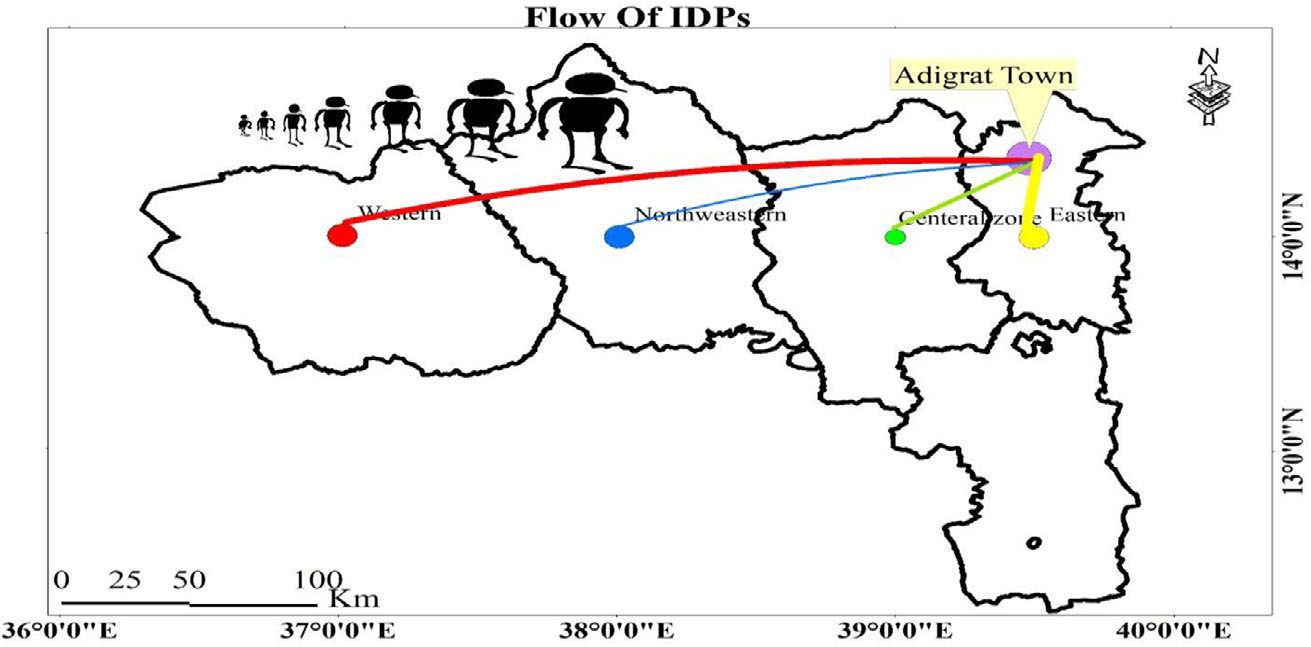
Flows of internal displaced peoples of the study. IDP, internally displaced person.

This outcome was further supported by a KII‐health expert, who noted: The destruction of the main hospital meant all complex cases were untreatable, leading to unnecessary deaths, even in routine maternity cases. Figure 3 illustrates movement from rural to urban areas and between urban centers in Tigray, converging in Adigrat City IDP centers.

### 3.2 Health Service Disruption

The findings from Table 5 indicate severe health service disruptions in IDP centers in Adigrat. The highest level of disruption was reported for destruction of health facilities (*M* = 4.98, SD = 0.13), followed by treatment of chronic diseases (*M* = 4.94, SD = 0.23) and shortage of drugs and supplies (*M* = 4.91, SD = 0.34). These results indicate widespread breakdown of essential health services during the conflict period.

**TABLE 5:** Health service disruption indicators in internally displaced person (IDP) centers.

Indicator	Mean	SD
Infant mortality increase	4.84	0.38
Maternal mortality increase	4.81	0.47
Elderly mortality increase	4.75	0.56
Decrease in life expectancy	4.83	0.45
Increase in birth rate	4.69	0.68
Family planning services decreased	4.87	0.38
Health extension services decreased	4.89	0.39
Shortage of drugs and supplies	4.91	0.34
Shortage of health professionals	4.86	0.39
Treatment of chronic diseases decreased	4.94	0.23
Institutional deliveries decreased	4.86	0.46
Destruction of health facilities	4.98	0.13

KIIs further supported these findings. A social affairs officer stated: “We saw people dying because of a lack of basic medication, not just violence….” A health expert added that the destruction of hospitals resulted in untreated emergency and maternity cases. On the basis of field observations, women and children were sleeping in tents without access to any medical treatment.

### 3.3 Sociodemographic and Contextual Determinants of Health Service Disruption

How has the war in Tigray affected the provision and quality of health services for IDPs in Adigrat City?

**FIGURE 4:**
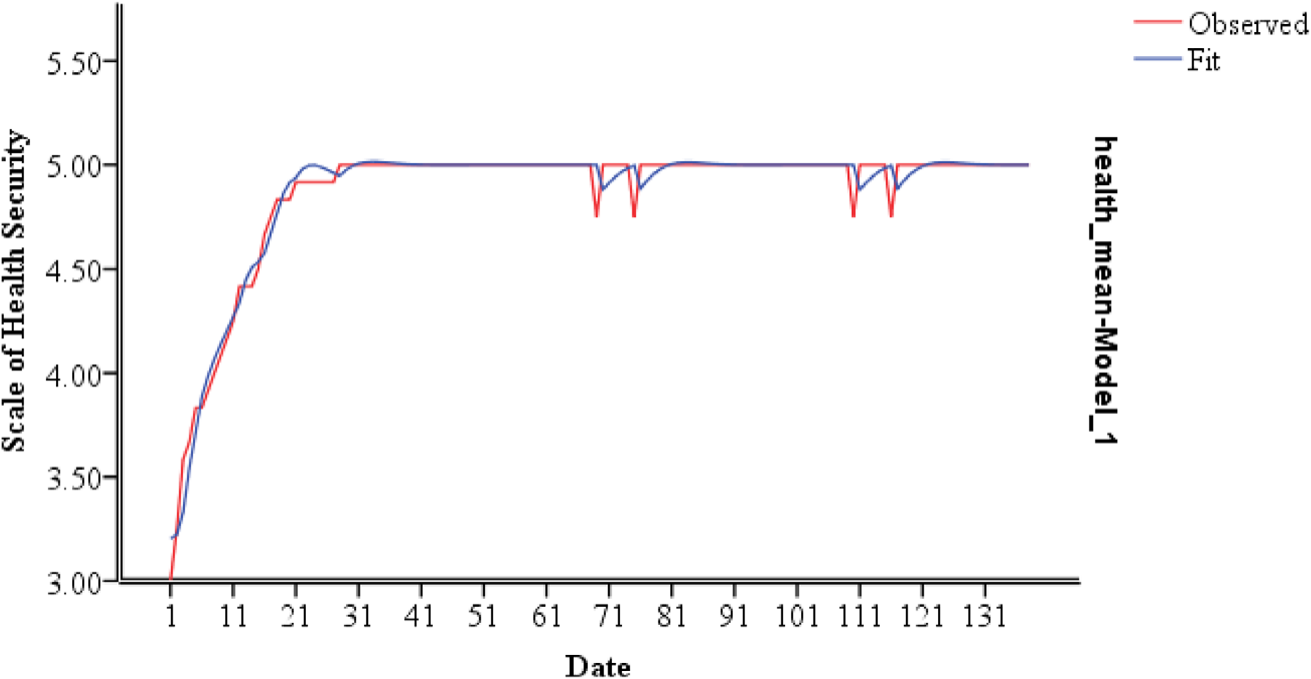
Forecast of health service disruption (2021–2023).

The analysis identifies both contextual (structural) and individual‐level (sociodemographic) factors as significant determinants of health service disruption (Figure 4).

#### 3.3.1 Regression Analysis

- Predictors: constant, shutdown of bank service, and crime mean.
- Dependent variable: health services and infrastructure index (mean disruption score).

Table 6 provides the model summary and coefficients of a simple linear regression analysis assessing the impacts of the shutdown of bank services and crime mean on health services and infrastructure. The model shows a strong relationship (*R* = 0.836, *R* square = 0.698) between the predictors (shutdown of bank services and crime mean) and the dependent variable (health services and infrastructure index). Both predictors have statistically significant coefficients (*p* < 0.001), indicating their impact on health services and infrastructure.

**TABLE 6:** Model summary of regression analysis.

Model	*R*	*R* square	Adjusted *R* square	Std. error	*R* square change
1	0.836	0.698	0.694	0.19618	0.698

The findings demonstrate that both crime and bank service disruptions significantly increase health service breakdown, with bank shutdowns having the strongest effect as indicated in Table 7.

**TABLE 7:** Regression coefficients.

Predictor	*B*	Std. error	Beta	*t*	*p*
Constant	1.232	0.208	—	5.928	0.000
Crime mean	0.172	0.034	0.279	5.089	0.000
Shutdown of bank services	0.578	0.048	0.659	12.008	0.000

The ANOVA results indicate that in Table 8, the regression model is statistically significant, *F*(2, 370) = 156.353, *p* < 0.001, suggesting that the predictors (crime and bank service shutdown) significantly explain variation in health service disruption.

**TABLE 8:** ANOVA results.

Model	Sum of squares	df	Mean square	*F*	*p*
Regression	12.035	2	6.018	156.353	0.000
Residual	5.196	370	0.038		
Total	17.231	372			

As Table 9 indicated, the time series model provides several goodness‐of‐fit statistics, with emphasis on the stationary *R*‐squared value. This statistic estimates the proportion of total variation in displaced periods explained by the model and is more suitable than ordinary *R*‐squared for series with trends or internal displacement patterns. A larger stationary *R*‐squared value (up to 1) indicates a better fit. A value of 0.719 suggests that the model effectively explains the variation in health service delivery and infrastructure affected by war‐related destruction and shutdowns. According to Hassani et al. [30], the Ljung–Box *Q* statistic tests if the model is correctly specified. A high *p* value (*p* > 0.05) indicates that the residuals are independently distributed (white noise), confirming correct specification. This model shows a strong positive association between observed data and model fit, highlighting the severe impact of war on health services and infrastructure. In line with this, the series chart clearly indicates a continuous decline in health service delivery alongside a rising pattern of disruption over time.

**TABLE 9:** Forecasting model of health service delivery (2021–2023).

Model	Stationary *R* ^2^	Ljung–Box *Q*	Outliers
Health model	0.719	0.984	226.502

### 3.4 Marginal Effects Analysis

According to Table 10, the marginal effects from the probit model (Pr [health services]) indicate the influence of key variables, including age, gender, humanitarian intervention, displacement, and war‐related factors, on access to health services.

**TABLE 10:** Marginal effects of health service determinants.

Variable	*dy*/*dx*	Std. error	*t* value	*p* value
Gender of household	0.1319	0.0198	6.67	0.000
Health status of IDPs	0.0080	0.0016	4.97	0.000
Health aid received	0.1291	0.0335	3.85	0.000
Number of IDPs	0.1352	0.0358	3.77	0.000
War intensity	−0.1629	0.0262	−6.22	0.000

Abbreviation: IDPs, internally displaced persons.

The results show that demographic factors significantly affect access to health services. Specifically, gender of HHs is positively associated with the likelihood of lacking health services, with a marginal effect of 0.1319, suggesting that HHs with higher gender‐related vulnerability are more likely to experience inadequate access. In addition, the health status of IDPs has a positive effect on the probability of lacking health services (marginal effect = 0.0080), indicating that individuals with poorer health conditions are more likely to face limited access to care.

### 3.5 Health Outcomes and Their Association With Health Service Disruptions

What are the key health outcomes among IDPs, and how are they associated with disruptions in healthcare services?

The study identifies worsening health outcomes among IDPs that are closely linked to disruptions in healthcare services.

**Humanitarian intervention factors**: Amount of health aid received: Higher amounts of health aid received increase the probability of health services being delivered (marginal effect = 0.1291).

**Displacement factors**: Number of displaced persons (IDPs): More displaced persons increase the probability of lacking health services (marginal effect = 0.1352).

**War‐related factors**: Intensity of the war: Increased war intensity decreases the probability of health services being delivered (marginal effect = −0.1629).

The analysis shows that increased displacement and war intensity worsen health service access, whereas humanitarian aid improves it.

## 4 Discussion

The findings of this study demonstrate that the war has had a severe and multifaceted impact on health service delivery in Adigrat City. Beyond the physical destruction of health infrastructure, the disruption of essential systems such as banking, transportation, and security significantly constrained access to healthcare services. These systemic breakdowns reduced the availability of medical supplies, limited service provision, and weakened the operational capacity of both healthcare facilities and humanitarian actors. A key contribution of this study is the demonstration that health system collapse in conflict settings is not driven solely by damage to health infrastructure but is strongly shaped by the simultaneous failure of interconnected financial and logistical systems. Although previous studies have documented the general relationship between armed conflict and health system deterioration [31, 32], this study provides more granular evidence from Adigrat City showing how banking shutdowns and transportation disruptions directly restricted humanitarian response capacity, including procurement, staffing, and supply distribution. This highlights a less‐examined but critical pathway through which conflict intensifies health service breakdown.

The findings also show that IDPs experienced particularly high vulnerability, reflected in elevated mortality and worsening health outcomes. These outcomes are driven not only by direct exposure to violence but also by indirect effects such as food insecurity, poor living conditions, and severely limited access to essential services. The study further underscores the importance of systemic and institutional factors—particularly financial infrastructure and security—in shaping healthcare access during armed conflict. The suspension of banking services significantly disrupted financial flows, limiting humanitarian organizations’ ability to function effectively. This finding expands current understanding by emphasizing that humanitarian effectiveness depends not only on physical access and security but also on the continuity of financial systems that enable operational delivery.

These results are consistent with ecological systems theory, which emphasizes that health outcomes are shaped by interactions across multiple interconnected systems. In the context of Adigrat, simultaneous disruptions across healthcare, financial, transport, and security systems created compounded vulnerabilities that intensified adverse health outcomes. Comparative evidence from other conflict‐affected settings supports these findings. In the Democratic Republic of Congo (DRC), prolonged conflict has contributed to persistently high child mortality rates [32]. Similarly, in Yemen, El Bcheraoui et al. [31] documented high burdens of diarrheal disease, anemia, and malnutrition among vulnerable populations, driven by disrupted health services, food insecurity, and inadequate water and sanitation systems. These cases reinforce the need for integrated humanitarian responses that address health, nutrition, water, and sanitation simultaneously.

Contemporary conflicts increasingly have disproportionate effects on civilians. Kravić [33] notes that modern civil conflicts often involve targeted violence against vulnerable populations, particularly women and children, resulting in large‐scale displacement. Historical evidence from Bosnia and Herzegovina (1992–1995) similarly demonstrates the severe health and social consequences of war, including mortality, injury, sexual violence, and long‐term psychosocial impacts such as orphan hood [34]. Recent evidence from Ukraine further confirms these dynamics. Ahsan [35] reports that healthcare systems in conflict‐affected areas have been severely disrupted due to infrastructure destruction, workforce displacement, and restricted access caused by insecurity. In besieged areas, health systems have largely collapsed, underscoring the fragility of service delivery under active conflict conditions.

## 5 Conclusion

The investigation utilized a combination of primary and secondary data sources, employing both qualitative and quantitative methodologies to comprehensively analyze the effects of war on IDPs, particularly focusing on health‐related outcomes. The primary data collection methods included questionnaires, field observations, and interviews, whereas secondary data were gathered from published and unpublished sources such as documents, journals, reports, and online resources.

The study findings underscore the profound impact of war on IDPs, particularly in terms of health outcomes. The research identified a range of detrimental effects, including increased infant mortality, maternal mortality, and mortality among elders. Additionally, the study observed an increase in birth rates alongside a decrease in family planning services and health extension services. The shortage of drugs, medical supplies, and health professionals in health centers and hospitals was also documented, further exacerbating the health crisis. Moreover, the destruction of health centers and hospitals due to war has severely compromised access to healthcare for IDPs.

Furthermore, the investigation highlighted the significant negative impact of the shutdown of bank services and increases in crime rates on health service delivery and infrastructure. The study employed forecasting models that demonstrated a strong correlation between observed and predicted values, confirming the consistency and reliability of the models used.

## 6 Recommendations

In order to decrease the negative impacts of war on IDP healthcare, the following recommendations are put forth, sorted by actor and viability:

Short‐term humanitarian response (international NGOs and donors are responsible actors):

- Expand essential health aid: Increase the amount and frequency of medical supplies, drugs, and treatments delivered to IDP camps. Boost vaccination efforts and mobile health clinics to give ongoing medical care even in the face of migration.
- Security and crime mitigation: Work with regional authorities and UN agencies to put security measures in place that decrease crime rates in camps and ensure secure channels for humanitarian supplies.

Long‐term system rebuilding (responsible actors: Ethiopian Ministry of Health [MOH], DONOrs):

- Infrastructure and personnel restoration: Set aside money to restore and enhance medical facilities and hospitals that were damaged or destroyed during the war. To address the substantial scarcity of medical staff, new health specialists should be educated and delivered quickly to IDP camps and reconstructed institutions.
- Targeted public health programming: Start initiatives to enhance maternal health, family planning, and child care. Educate IDP populations on managing chronic illnesses (such as diabetes and hypertension) and preventive healthcare practices.

Policy and advocacy (responsible actors: MOH, regional government): Promote laws that safeguard the rights and welfare of IDPs, such as continued access to social services and healthcare, and carry out continuous monitoring to evaluate changing health requirements and modify interventions as necessary.

In conclusion, addressing the complex health challenges faced by IDPs requires a multifaceted approach that includes immediate humanitarian aid, long‐term health system strengthening, and advocacy for peace and stability. By implementing these recommendations, stakeholders can work toward improving health outcomes and rebuilding resilient health systems for internally displaced populations affected by war.

## Author Contributions

**Fikre Belay Tekulu**: writing – original draft, conceptualization, data curation, writing – review, and editing. **Haftom Teshale Gebre**: writing – original draft, conceptualization, data curation, writing – review, and editing. **Meseret Meresa Weldemichael**: data curation. **Esayas Weldetinsae Gebrekidan**: data curation.

## Ethics Statement

Approval was obtained from the Institutional Review Board (IRB) of Adigrat University (approval number: AU/IRB/007/2023). Verbal informed consent was obtained from all participants. Narrative data used pseudonyms to maintain confidentiality. De‐identified quantitative data and qualitative codebooks are available from the corresponding author upon reasonable request.

## Conflicts of Interest

The authors declare no conflicts of interest.

## Data Availability

All data generated or analyzed during this study are included in this. Further inquiries can be directed to the corresponding author.
